# Resistant Ventricular Tachycardia due to Idiopathic Left Ventricular Aneurysm: Successful Treatment with Surgery

**Published:** 2019-01

**Authors:** Muhammed Keskin, Tufan Çınar, Mert İlker Hayıroğlu, Ömer Kozan

**Affiliations:** *Sultan Abdülhamid Han Training and Research Hospital,* *Health Sciences University, Istanbul, Turkey.*

**Keywords:** *Heart ventricles*, *Aneurysm*, *Tachycardia, ventricular*, *Surgical procedures, operative*

## Abstract

Left ventricular aneurysms (LVAs) are characterized by a wide connection to the left ventricle and paradoxical systolic motions. Although patients with LVAs are usually asymptomatic, some may present with arrhythmias, heart failure, and even cardiac arrest. In this case report, we describe a 62-year-old male patient who presented to our emergency service with complaints of palpitation and shortness of breath of 2 hours’ duration. His blood pressure was 84/56 mm Hg, and he was in a confused state. An electrocardiogram revealed ventricular tachycardia (VT) with right bundle branch block and a ventricular rate of 188 bpm. The patient’s hemodynamic instability necessitated a direct current cardioversion, which restored the sinus rhythm. During the in-hospital course, he had numerous recurrent VT episodes despite treatment with intravenous amiodarone and magnesium sulfate as well as radiofrequency ablation. Upon consensus with a cardiovascular surgeon’s team, urgent surgery was performed due to the resistant VT episodes. The patient’s clinical course was uneventful, and he was discharged on the 11th postoperative day. We have been following up the patient for almost 1 year, during which he has not experienced palpitations or associated symptoms. Our case indicates that surgery may be a preferable treatment option for patients with heart failure and resistant VT related to LVAs.

## Introduction

According to a recent article by Xia et al.,^1^ a left ventricular aneurysm (LVA) is an area of an abnormal left ventricular (LV) diastolic contour with systolic dyskinesia or paradoxical bulging, while Ruzza et al.^2^ defined an LVA as an area of the distinct akinesia or dyskinesia of the LV wall. LVAs most frequently develop after a myocardial infarction. Idiopathic LVAs, which do not have an identifiable underlying cause, are very rare. In this report, we present the case of a resistant ventricular tachycardia (VT) related to an LVA, which was treated successfully with surgical aneurysmectomy.

## Case Report

**Figure 1:**
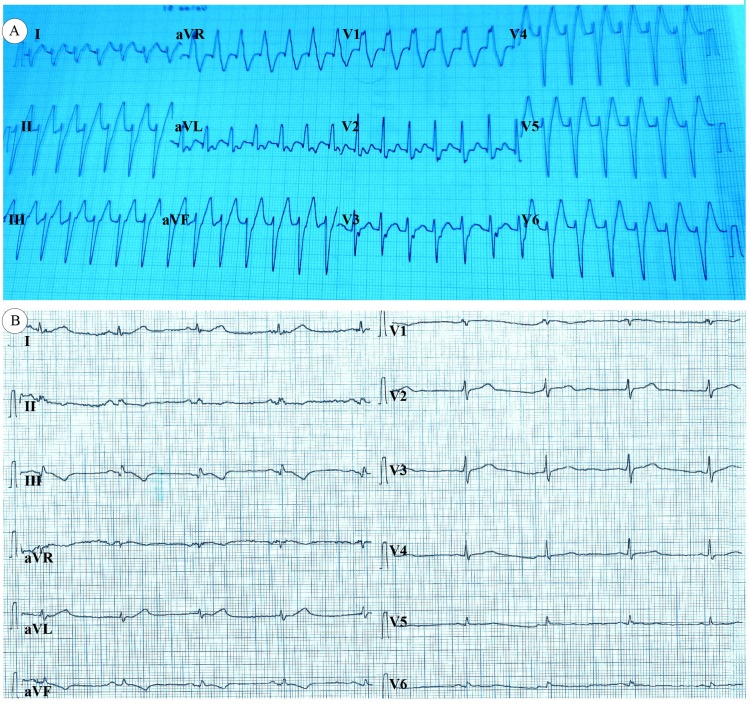
Electrocardiograms of the patient: A) electrocardiography on admission to the emergency department, showing ventricular tachycardia with the right bundle branch block configuration and a superior axis; and B) post-cardioversion electrocardiography, showing a normal sinus rhythm

**Figure 2:**
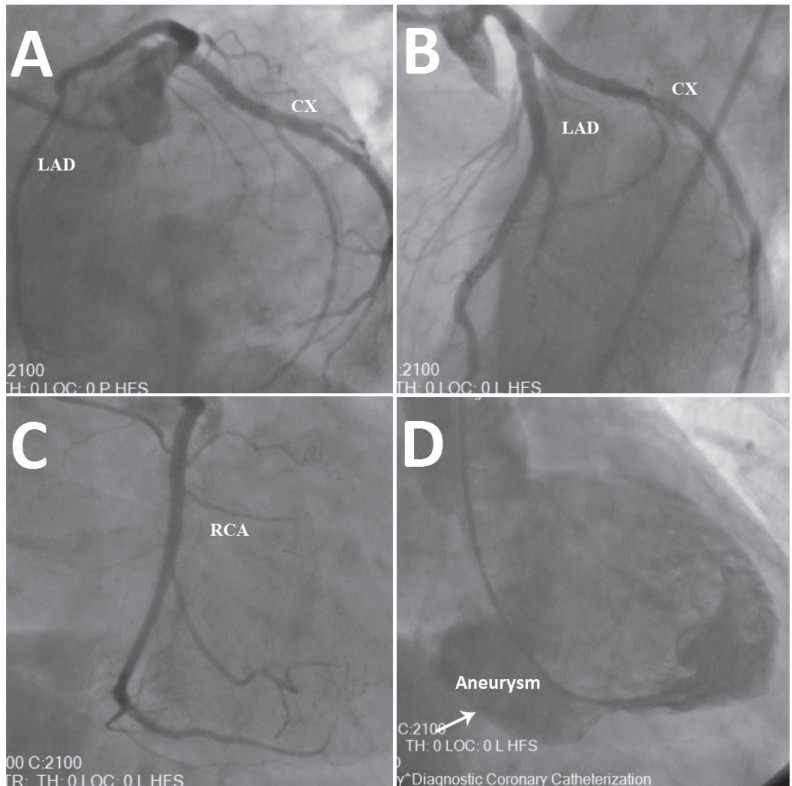
Coronary angiographic studies of the patient: A) left anterior oblique caudal view, revealing a patent left descending anterior coronary artery (LAD) and circumflex artery (CX) without thrombus, dissection, or coronary slow flow; B) left anterior oblique cranial view, revealing a patent LAD and CX without thrombus, dissection, or coronary slow flow; C) right anterior oblique view, revealing a patent right coronary artery (RCA) without thrombus, dissection, or coronary slow flow; and D) left ventriculogram obtained from the right anterior oblique view, showing the true left ventricular aneurysm (arrow) on the posterobasal segment of the left ventricle

A 62-year-old male patient presented to our emergency service with complaints of palpitation and shortness of breath of 2 hours’ duration. The patient’s relatives reported that he had no known diseases. His blood pressure was 84/56 mm Hg, and he was in a confused state. Cardiac and pulmonary auscultations revealed third heart sounds and bilateral fine crackles in the basal area of the lungs. An electrocardiogram (ECG) revealed VT with right bundle branch block and a ventricular rate of 188 bpm. The analysis of the ECG showed a superior axis. The use of direct current cardioversion restored the sinus rhythm (Figures 1A and 1B) and improved the patient’s blood pressure and hemodynamic state. Transthoracic echocardiography showed that the LV ejection fraction was 40% and that there was a large echolucent space (4.6 cm × 3.7 cm in diameter) on the posterobasal portion of the LV. The LV wall motion was normal, with the exception of the LVA segment. The wall thickness of the aneurysm sac was similar to the normal thickness of the LV wall. Laboratory analysis showed a slight elevation in creatine kinase-myocardial band (23 IU/L) and troponin I (0.12 ng/mL). Coronary angiography was performed via the femoral approach, and it demonstrated patent coronary arteries with no thrombus, dissection, or coronary anomaly. However, cardiac ventriculogram confirmed that the position of the aneurysm was on a posterobasal segment (Figures 2). 

The patient was diagnosed with an idiopathic LVA and treated with an angiotensin-converting enzyme inhibitor, a beta-blocker, and an aldosterone-receptor blocker. Because the VT reoccurred during hospitalization, intravenous (IV) amiodarone was administered. Moreover, an IV bolus dose of magnesium sulfate (up to 3 gr) was given. Despite the treatment with IV amiodarone and magnesium sulfate, the patient had numerous recurrent VT episodes. However, when IV lidocaine was added to the current therapy, the frequency of the VT episodes slightly decreased. Because the patient’s hemodynamic state was deteriorating during some of the VT episodes, direct current cardioversion was conducted several times. An electrophysiological study was performed using 3D mapping with the EnSite Precision cardiac mapping system (St. Jude Medical), and it revealed that the VT was associated with scar formation and the tachycardia cycle length was 390 ms. Nonetheless, radiofrequency ablation did not terminate the VT. Upon consensus with a cardiovascular surgeon’s team, urgent surgery was performed due to the resistant VT episodes. The patient underwent cardiac surgery, during which the idiopathic LVA was removed and repaired with a Dacron graft. The patient’s clinical course was uneventful. Because the patient had no known diseases, we investigated the immunologic and infective causes associated with the LVA such as sarcoidosis, Chagas disease, and syphilis; all the results were, however, negative. The patient was discharged on the 11th day after the operation. At the time of this report, we have been following up the patient for almost 1 year; he has not experienced palpitations or associated symptoms during this period. Finally, the patient underwent a 72-hour Holter monitoring, which showed no non-sustained or sustained VT episodes.

## Discussion

LVAs are usually the consequence of coronary artery disease, although they may also occur with congenital, traumatic, connective tissue, primary myocardial, or infective heart diseases. LVAs can be complicated by heart failure, thrombus formation, peripheral embolism, cardiac rupture, ventricular arrhythmias, or cardiac arrest.^3^ Klein et al.^4^ indicated that when approximately 20% to 25% of the LV area is inactivated by any pathological process, the degree of shortening distance required by the myofibril to maintain the stroke volume exceeds physiological limits and, thus, cardiac enlargement occurs to sustain an adequate ejection of blood. However, there is no relationship between heart failure and cardiac enlargement, as normal or nearly normal-sized hearts do not preclude heart failure. Management strategies in patients with idiopathic LVAs associated with VT include antiarrhythmic drug treatment, the implantation of a cardioverter defibrillator, catheter ablation, and surgical aneurysmectomy. The 3 main indications for surgery are angina (40%), cardiac failure (35%), and arrhythmias (10%).^5^^, ^^6^ Rozanski et al.^7^ investigated the ECG of VT in patients with LVAs and concluded that after a surgical aneurysmectomy, the ECG findings had changed. Accordingly, we can state that surgery removes the segment that triggers VT. It has been demonstrated that with specific patient selection, aneurysmectomy can improve symptomatic patients and has excellent 5-year survival rates.^8^ VT may also be responsive to catheter ablation.^9^ However, because VT can reoccur after ablation, another treatment may be required. Thus, there is no consensus on which treatment option is superior. Therefore, more research should be performed to compare catheter ablation with surgical aneurysmectomy.

## Conclusion

Our case indicated that surgery may be a preferable treatment option for patients with heart failure and resistant ventricular tachycardia related to left ventricular aneurysms. Hence, more research should be performed to compare catheter ablation with surgical aneurysmectomy in terms of success among these patients.

## Notes:

***This paper should be cited as: ***Keskin M, Çınar T, İlker Hayıroğlu M, Kozan Ö. Resistant Ventricular Tachycardia due to Idiopathic Left Ventricular Aneurysm: Successful Treatment with Surgery. J Teh Univ Heart Ctr 2019;14(1):33-36.
